# Factors associated with cervical cancer screening in the South African demographic and health survey

**DOI:** 10.1007/s10552-026-02205-5

**Published:** 2026-06-30

**Authors:** M. G. Singini, F. Sitas, P. Basu, A. P. Kengne, N. Peer

**Affiliations:** 1 Non-Communicable Diseases Research Unit, South African Medical Research Council, Cape Town and Durban, South Africa; 2 International Centre for Future Health Systems and School of Population Health, University of New South Wales, Sydney, Australia; 3 Burden of Disease Research Unit, South African Medical Research Council, Cape Town, South Africa; 4 Early Detection, Prevention & Infections Branch, International Agency for Research on Cancer (WHO), Lyon, France; 5 Department of Medicine, University of Cape Town, Cape Town, South Africa; 6 African Population and Health Research Center, Nairobi, Kenya

**Keywords:** Cervical cancer, Screening, South Africa, Empowerment, Disparities

## Abstract

**Introduction:**

Cervical cancer is South Africa’s second most common cancer among women, yet for complex reasons, Papanicolaou screening uptake remains suboptimal. Only a few studies have explored the interplay between sociodemographic characteristics, women’s empowerment, healthcare access, and lifestyle behaviors in influencing screening participation. We examined the associations between cervical cancer screening uptake and sociodemographic, women’s empowerment, healthcare access, and risky lifestyle behaviors factors.

**Methods:**

Cross-sectional analyses of a nationally representative dataset of women (*n* = 2789) aged 25–49 years were conducted from the 2016 South African Demographic and Health Survey. We used multivariable generalized linear regression models with Poisson distribution to estimate screening uptake (adjusted prevalence) ratios (aPRs, and 95% confidence intervals (95% CI)). We ordered the factors by their attributable fractions (AF), which takes the proportion of uptake per category into account.

**Results:**

Being tested for HIV (aPR = 1.79; 95% CI:1.40–2.30; AF = 41.7%), population group (being Black) (aPR = 0.75; 95% CI:0.66–0.84; AF = 19.7%), rich (aPR = 1.34, 95% CI:1.08–1.67; AF = 15.9%), and education (aPR = 1.18, 95% CI:1.03–1.36; AF = 8.9%), were the most important determinants of cervical screening uptake in South Africa. Alcohol misuse (aPR = 1.23; 95% CI:1.10–1.38; AF = 6.1%), having health insurance (aPR:1.27; 95% CI:1.12–1.45; AF = 5.5%) and disempowerment (aPR = 1.10; 95% CI:1.00–1.22; AF = 5.5%) were less important drivers of screening uptake.

**Conclusions:**

Pap smear uptake remains suboptimal, particularly among Black women. There is a need for enhanced awareness and education initiatives as well as novel strategies involving community-based screening programs to encourage the uptake of cervical screening.

**Supplementary Information:**

The online version contains supplementary material available at 10.1007/s10552-026-02205-5.

## Background

Cervical cancer remains one of the most preventable yet persistently burdensome cancers among women globally with 660, 000 new cases estimated worldwide in 2022 [1]; notably, 90% of cases occur in low- and middle-income countries (LMICs) [1–3]. In South Africa, cervical cancer remains a major public health concern, ranking as the second most common malignancy among women. The overall age-standardized incidence rate (ASIR) was estimated at 30.2 per 100,000 women in 2022. However, substantial disparities exist across population groups with ASIRs of 19.8, 19.5, 10.5, and 35.2 per 100,000 among White, Mixed ancestry, Indian/Asian, and Black African women, respectively [4]. These differences may reflect underlying inequities in exposure to human papilloma virus (HPV), access to healthcare and screening services, and socioeconomic status [4–6].

Early detection through quality-assured Papanicolaou (Pap) screening is critical for reducing incidence and mortality of cervical cancer [7]. Local studies in South Africa demonstrate that increase in uptake of cervical cancer screening programs using Pap smears could contribute to achieving the national cervical cancer screening target of 70% [5] from the current suboptimal prevalence of 42% [8]. Globally, only a few studies have explored the complex interplay between sociodemographic characteristics, women’s autonomy, healthcare access and lifestyle behaviors in influencing screening participation. Even then, these dimensions are often treated in silos rather than within a unified or integrated analytical framework [9–15]. While previous studies in South Africa have identified individual-level factors such as age, education, population group, and HIV status as important determinants of cervical cancer screening [7, 14, 15], most have examined these factors in isolation. In particular, the role of women’s autonomy and socioeconomic empowerment, critical drivers of health-seeking behaviors [13, 16] have been underexplored in the context of cervical cancer screening in South Africa. Understanding how these multifactorial influences interact is critical for designing targeted interventions that address both structural and behavioral barriers to screening uptake.

The aim of this study was to investigate factors associated with the uptake of cervical cancer screening among women aged 25–49 years in South Africa, using nationally representative data. This was examined by disaggregating predictors across four domains of sociodemographic factors, viz. 1) female autonomy, 2) socioeconomic status, 3) risky lifestyle behaviors, and 4) healthcare access, to capture the multiple pathways influencing screening uptake and to examine a broader range of social determinants of health in cervical cancer screening.

## Methods

### Study design and population

Data for this secondary analysis were obtained from the 2016 South African Demographic and Health Survey (SADHS-2016), a nationally representative cross-sectional survey. The analysis was restricted to women aged 25–49 years, in line with WHO [17] cervical cancer screening guidelines. A two-stage stratified cluster sampling design was employed in the survey. In the first stage, enumeration areas (EAs) were selected using probability proportional to size sampling, followed by systematic sampling of households within selected EAs in the second stage [18].

### Data collection

Data for the SADHS-2016 were collected from eligible participants using standardized, pre-tested, and interviewer-administered questionnaires. In the SADHS-2016, data collection procedures, training of fieldworkers, and quality assurance protocols were implemented using standardized techniques and are described elsewhere [18]. For this analysis only data collected using the women questionnaire were used.

### Outcome variable

The outcome variable was self-reported cervical cancer screening this was defined by having ever had a Pap smear. The participants were asked if they ever had a Pap smear. The response to the question were recorded in binary format as “yes” and “no.” Those who responded “don’t know” were excluded from the analysis.

### Variables assessed

Participants aged less than 25 years were excluded. The following sociodemographic categorical variables were employed: age grouped in years 25–29 (reference category), 30–39, 40–49), education (primary (< = 7 years), secondary (8–12 years) and tertiary), marital status (not married, married, other (widowed, divorced, no longer living together/separated)), place of residence (rural, urban), employment status (Yes/No), quintiles of wealth (1(lowest), 2, 3, 4, 5 (highest)) which were used to define wealth status, and health insurance coverage (yes or no). The clinical variables were whether they were HIV tested (Yes/No), HIV test result (negative, positive), smoking frequency (no, or occasional/daily smokers, i.e., >  = 1 cigarette/day), regular contraceptive use (no, oral/injectable and others), alcohol misuse (no, yes, as measured by the CAGE set of four questions [19], number of sexual partners (0,1,2 +), parity (0–1, 2, 3 +), having children aged < 5 years (no, yes). An empowerment index was generated by summing six decision‑making variables (related to contraceptive use, accessing healthcare, large purchases, visiting relatives, and use of husband’s earnings [20], each coded as 1 if the woman was involved in the decision (alone, jointly, or with someone else) and 0 otherwise. Based on the total score (0–6), women were categorized as not empowered (0–2), less empowered (3–4), or highly empowered (5–6).

### Statistical analysis

Statistical analyses were conducted using Stata version 15 (Stata Corp, College Station, TX, USA) and R (version 4.4.3). All analyses incorporated the survey design of the DHS, including sampling weights, primary sampling units (PSUs), and stratification variables to ensure nationally representative estimates and accurate standard errors. Descriptive analyses of individual characteristics were presented as absolute numbers and percentages. Differences between groups were assessed using chi-square (χ2) tests for categorical variables and the F-statistic for ordinal variables. We examined disparities in cervical cancer screening by assessing the distribution of screening prevalence across wealth quintiles within each population group. Weighted prevalence estimates were calculated for each combination of wealth index and population group, accounting for the complex survey design (Table S1). The estimates were visualized using stratified line graphs, with separate panels for each population group. We assessed the prevalence of Pap smear uptake for cervical cancer screening across age groups, stratified by recommended screening intervals.

We used a generalized linear regression model (GLM) with a binomial family and logit link to examine associations between empowerment (empowered vs. not empowered), population group (other population groups vs. Black), wealth status (poor vs. rich), and cervical cancer screening, adjusting for age. Two- and three-way interactions between empowerment, population groups, and wealth status were included. We examined relative inequalities in cervical cancer screening across socioeconomic strata and population groups. These estimates were visualized using faceted line plots, with each panel representing a different population group. Predicted probabilities for all combinations of empowerment, population groups, and wealth status were calculated from the fitted model while holding age at its mean. Estimates were transformed from the logit to the probability scale, with 95% confidence intervals derived from standard errors. Predicted probabilities were visualized using bar plots to illustrate the joint effects of sociodemographic factors on screening uptake. We assessed collinearity of variables using Variance Inflation Factor (VIF).

To assess factors associated with cervical cancer screening GLMs with a Poisson distribution and log-link function were employed to estimate adjusted prevalence ratios (aPRs) and 95% confidence levels for cervical cancer screening uptake [21–23]. This modeling strategy was selected to accommodate the cross-sectional nature of the data and the relatively high prevalence of the outcome, for which odds ratios may overstate associations [23]. The quasi-Poisson specification accounts for overdispersion by modifying the variance function, yielding robust standard error estimates and improving inference reliability [21].

## Four different models were conducted

### The first model was the basic model (sociodemographic)

Variables included were age (years), population group (Black African, White, Mixed ancestry and India/Asian), wealth index (quintile from 1 lowest to 5 highest) and education level (primary, secondary, tertiary). In the second model (women autonomy), the variables included were women empowerment status, employment while adjusting for all the variables in the basic model. The third model (healthcare access) had the following independent variables: HIV tested (ever), place of residence (rural/urban), health insurance coverage, regular use of contraception, parity and having children aged < 5 years while adjusting for the basic model. The fourth model (lifestyle risky behaviors) included the following variables: smoking frequency, alcohol misuse, and number of current sexual partners, while adjusting for the sociodemographic variables in the basic model.

We estimated the attributable fractions (AF), assuming there was a causal relationship between the predictors and cervical cancer screening uptake; AF were estimated as an increase in cervical cancer screening uptake that would occur if women in the study shifted to the adjacent healthier category of cervical cancer screening uptake.

We calculated the number of women screened in each category (Si) as:

$${S}_{i}=\left(\frac{{P}_{e,i}}{100}\right)*{N}_{i}$$

where.

${S}_{i}$= number of women screened in each category.

${P}_{e,i}$= prevalence of screened (%) in category i.

${N}_{i}$= total number of women in that category.

We estimated the AF among the exposed (AFE) assuming everything is causal.

$${AFE}_{i}=1-\frac{1}{{aRR}_{i}}$$

And the corresponding AF of women screened as.

$${Attributable fraction (AF)}_{i}={S}_{i} x(1- \frac{1}{a{RR}_{i}})$$ [24].

For variables with more than two categories, we obtained the total attributable number by summing the category-specific values. We used APR as an approximation of Relative Risk. Table S2 provides details of ${aPR}_{i}$ and ${AF}_{i}$,

We conducted a sensitivity analysis using forward stepwise regression with Akaike information criterion (AIC)-based selection (unweighted Poisson for variable selection, followed by refitting with survey-weighted modified Poisson). We retained into the final model variables which were excluded by the stepwise procedure such as women’s empowerment, education level, and population group, but are well established in the literature and were significant in at least four other models.

### Ethical approval

The South African Medical Research Council Ethical Committee approved the SADHS protocol. Informed consent was obtained from all participants.

## Results

In 2016, the SADHS recruited 8,514 women from all nine provinces in South Africa. The final weighted analytic sample comprised 2,789 women aged 25–49 years (Table 1). Of these, 1,152 (41.3%) were aged 30–39 years. Most participants identified as Black African (85.9%). Over 80% had attained at least secondary education, 46% of participants were unmarried, 45% were employed, and 17% had private health insurance (Table 1).

**Table 1:** Uptake for cervical cancer screening using Pap smear, by sociodemographic, autonomy, healthcare access, and risky lifestyle behavior of the study participants of women aged 25–49 years

Characteristics	Total (*n* = 2,789)	Percentage (%)	Screened for cervical cancer *n* = 1,261 (45.2%)	Not screened for cancer *n* = 1,528 (54.8%)	*p*-value
Sociodemographic	*n*		*n* (%)		
Age group (years)					** < 0.001**
25–29	729	26.1	215 (29.6)	513 (70.4)	
30–39	1,153	41.3	535 (46.4)	618 (53.6)	
40–49	908	32.6	511 (56.3)	397 (43.7)	
Population group					** < 0.001**
Black	2,396	85.9	982 (41.0)	1,413 (59.0)	
White	105	3.8	81 (76.7)	25 (23.4)	
Mixed ancestry	239	8.6	167 (70.0)	72 (30.0)	
Asian	50	1.8	31 (64.8)	19 (35.2)	
Wealth index (quintiles)					** < 0.001**
1 (lowest)	498	17.9	158 (31.8)	340 (68.2)	
2	536	19.2	187 (34.9)	349 (65.1)	
3	617	22.1	258 (41.7)	359 (58.1)	
4	569	20.4	287 (50.5)	282 (49.5)	
5 (highest)	569	20.4	370 (65.0)	199 (35.0)	
Education (years)					** < 0.001**
Primary or less (< = 7 years)	381	13.6	132 (34.8)	248 (65.2)	
Secondary (8–12 years)	1,209	43.3	505 (41.8)	703 (58.2)	
Higher	1,200	43.0	623 (51.9)	577 (48.1)	
Marital status					** < 0.001**
Not married	1,279	45.9	501 (39.1)	778 (60.9)	
Married	1,286	46.1	656 (51.0)	630 (49.0)	
*Other	224	8.0	105 (46.6)	120 (53.4)	
Women Autonomy					
#Women empowerment status					** < 0.001**
Not empowered	1,552		629 (40.1)	923 (59.5)	
Less empowered	329		140 (42.5)	189 (57.5)	
Highly empowered	909		493 (54.2)	416 (45.8)	
Employment status					** < 0.001**
No	1,526		610 (40.1)	916 (60.0)	
Yes	1,263		651 (51.5)	613 (48.5)	
Healthcare access					** < 0.001**
HIV tested ever					
No	264		69 (26.2)	195 (73.8)	
Yes	2,526		1,192 (47.2)	1,334 (52.8)	
Health insurance coverage					** < 0.001**
No	2,319		935 (40.3)	1,385 (59.7)	
Yes	470		326 (69.4)	144 (30.6)	
Place of residence					** < 0.001**
Urban	1,901		937 (49.3)	964 (50.7)	
Rural	888		324 (36.5)	564 (63.5)	
Parity					0.335
0–1	919		392 (42.7)	527 (57.3)	
2	825		392 (47.5)	433 (52.5)	
3 +	1,045		477 (45.6)	568 (54.4)	
Having children aged < 5 years					** < 0.001**
No	1,387		704 (50.7)	683 (49.3)	
Yes	1,402		557 (39.7)	845 (60.3)	
Regular use of contraceptive					0.150
No	1,348		593 (44.0)	755 (56.0)	
Oral/injectable	780		340 (43.6)	440 (56.4)	
Other	659		326 (49.5)	333 (50.5)	
Risky Lifestyle Behaviors					
Smoking frequency					** < 0.001**
No or occasional smokers	2,626		1,159(44.1)	1,467(55.9)	
> = 1 cigarette per day	163		102 (62.7)	60.9 (37.3)	
alcohol misuse					** < 0.001**
No (CAGE = 0–1)	2,041		847 (41.5)	1,194(58.5)	
Yes (CAGE > = 2)	748		414 (55.3)	334 (44.7)	
Number of current sexual partners					** < 0.001**
0	1,603		799 (49.8)	805 (50.2)	
1	1,113		434 (39.0)	679 (61.0)	
2 +	72		28 (39.1)	44 (60.9)	

*,#See methods

The results statistically signifcant at *p*<0.05

**Fig. 1:**
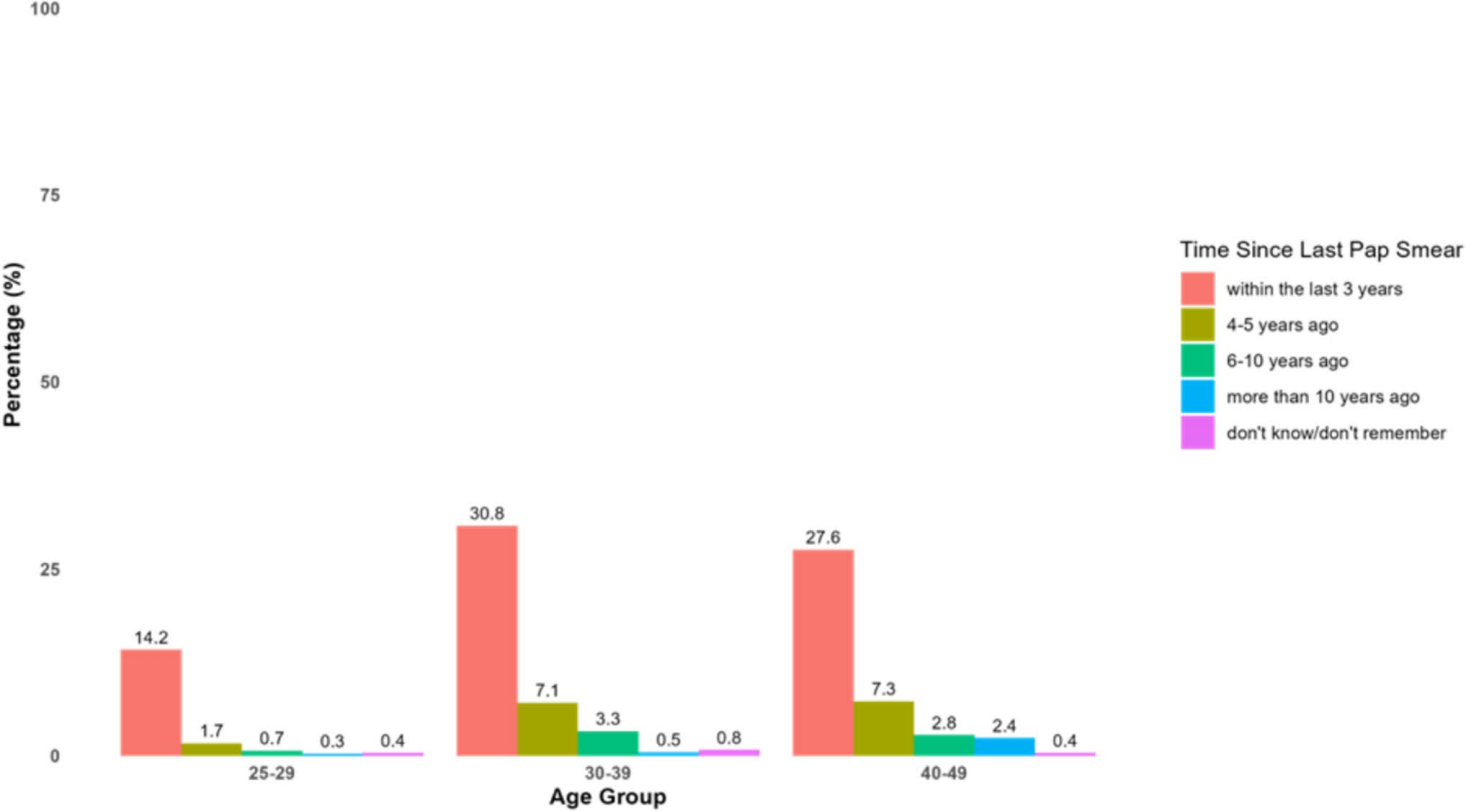
Period of Pap smear uptake for cervical cancer screening by age group

Overall, 45.2% of women in this study reported ever having a Pap smear uptake for cervical cancer screening (Table 1). Among those who reported screening, most (72.6%) had been screened within the previous 3 years (Fig. 1). Screening uptake varied significantly by age group, with the highest uptake among women aged 40–49 years (56.0%) and the lowest among those aged 25–29 years (29.6%; *p *< 0.001; Table 1). Black African women had the lowest Pap smear screening uptake (41.0%), compared with White women (76.7%), women of Mixed ancestry (70.0%), and Indian/Asian women (64.8%) (*p* < 0.001). Screening uptake was also higher among women in the highest wealth quintile than among those in the lowest (65.0% vs. 31.8%; *p* < 0.001). Women with health insurance had higher Pap smear uptake than those without insurance (69.4% vs. 30.6%; *p* < 0.001). Interestingly, current smokers had higher Pap smear uptake than non-smokers or occasional smokers (62.7% vs. 44.1%; *p* < 0.001) (Table 1).

**Fig. 2:**
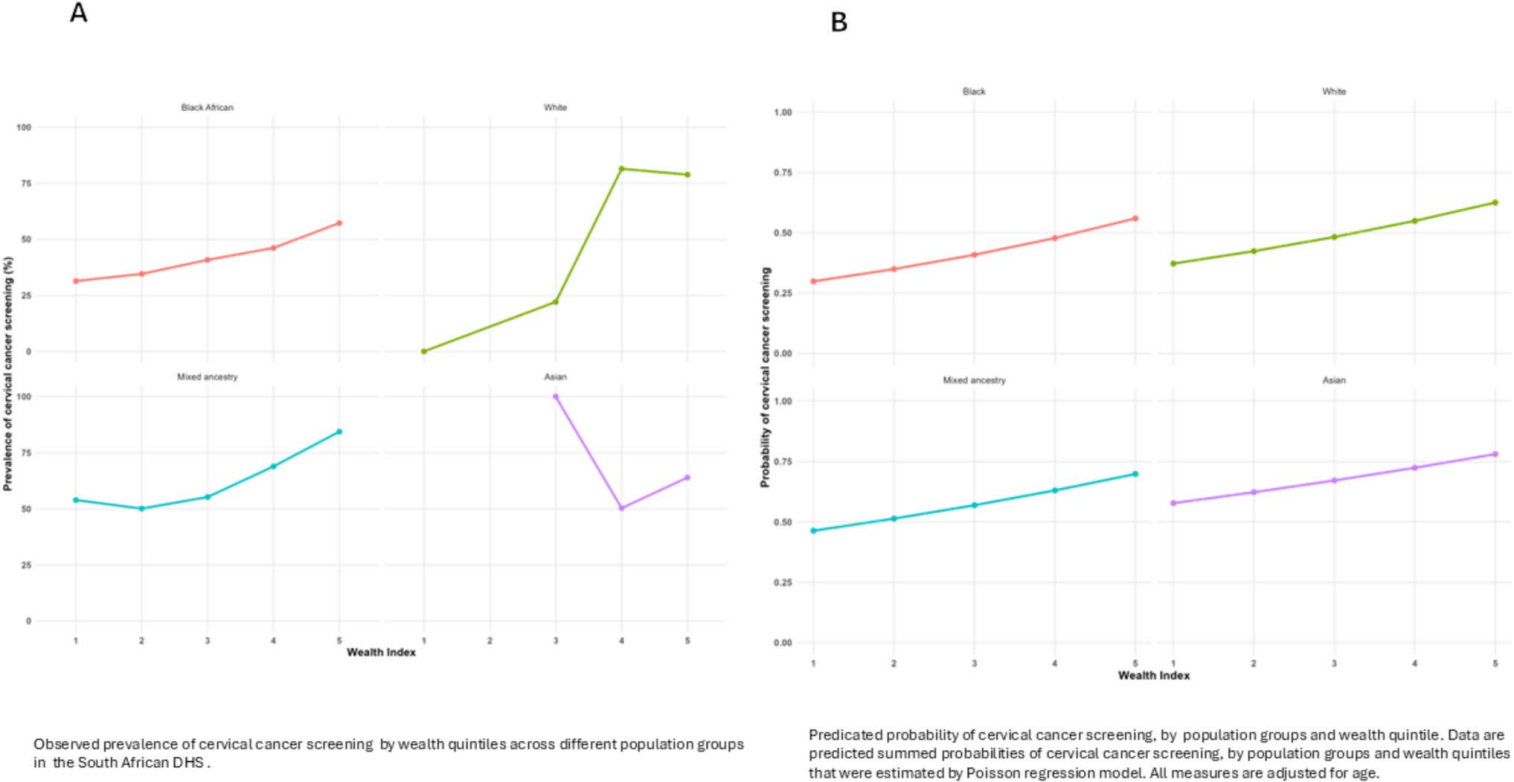
Observed and predicated disparities in cervical cancer screening uptake in South Africa by wealth index and population groups

Survey-weighted estimates Pap smear uptake for cervical cancer screening prevalence rates revealed notable disparities across different wealth quintiles and population group. Among Black African women, Pap smear uptake prevalence rates were low 25.0% in the lowest wealth quintile to 55.0% in the highest. White women showed a sharp rise from 40.0 to 60.0% between the lowest and middle quintiles, followed by a slight decline at higher wealth levels. Women of Mixed ancestry exhibited the highest overall prevalence rates, from 50.0 to 75.0% across the wealth spectrum. Asian women showed moderate increases, with prevalence ranging from 45.0 to 65.0% (Fig. 2A). A disproportionately higher percentage of women who reported undergoing cervical cancer screening and possessing health insurance was White (78.4%) compared to Black women (20.0%) Table S2. Women of Mixed ancestry had significantly higher predicted probabilities of screening compared to Black African women (*p* = 0.009), while White women had significantly lower probabilities (*p* < 0.001). Age also played a significant role, with women aged 30–39 and 40–49 showing higher predicted probabilities of screening compared to those aged 25–29 (*p* < 0.001 for both) (Fig. 2B).

**Fig. 3:**
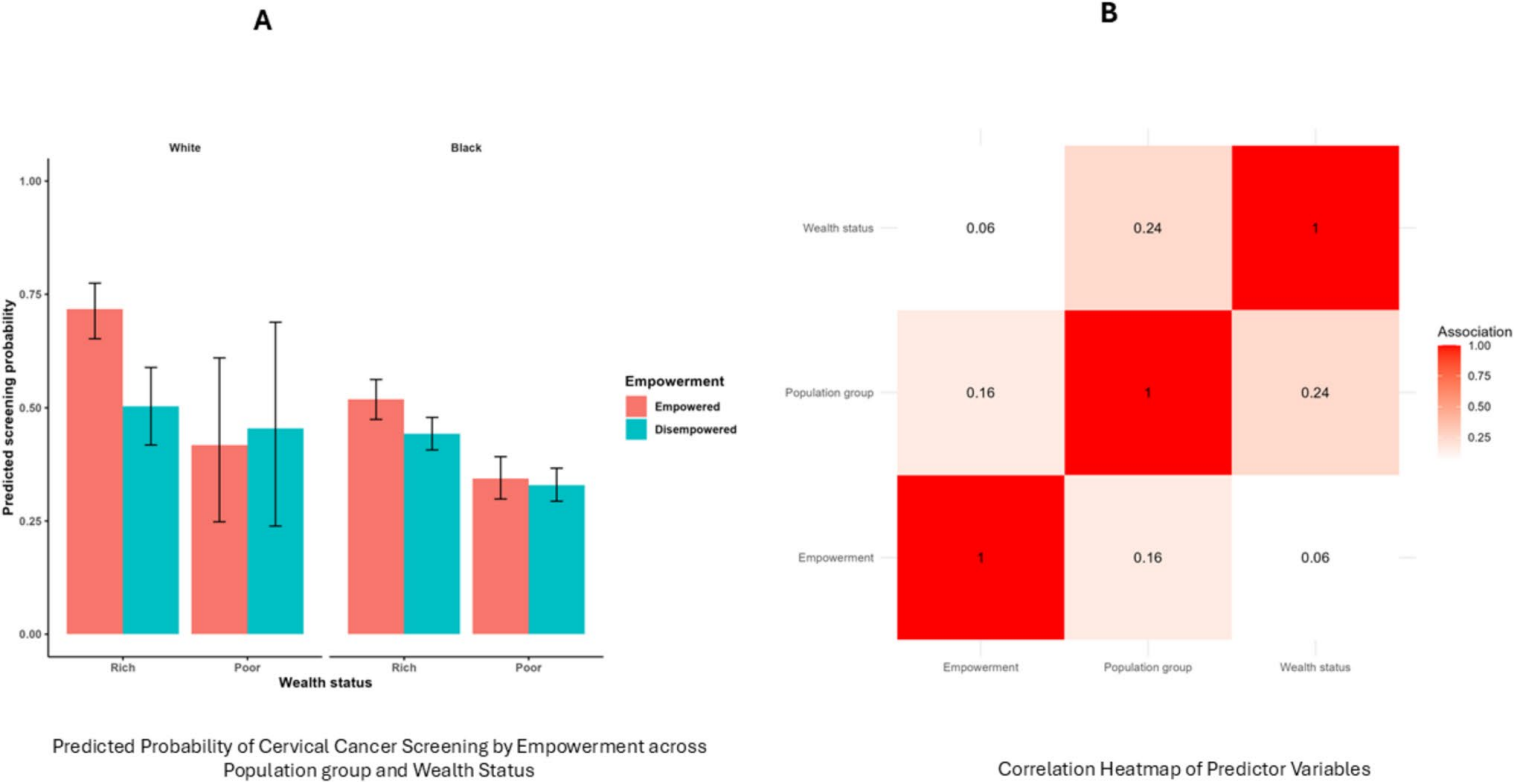
**A** Predicted probability of cervical cancer screening by empowerment across population group and wealth status. **B** Checking for collinearity across predicted variables using a correlation Heatmap

In the adjusted GLM, we did not observe any statistically significant main effects or interaction terms for empowerment status, population group, or wealth status in relation to cervical cancer screening uptake (all *p*-values > 0.05). However, model-based predicted probabilities suggested potential patterns of interest. Among White women, those empowered and not poor had the highest estimated probability of screening, while non-empowered and poor individuals had the lowest. These differences were less pronounced among Black women, where predicted probabilities varied minimally across empowerment and wealth strata. Confidence intervals around the predicted probabilities were wide, reflecting uncertainty in the estimates and reinforcing the need for cautious interpretation. The results VIF (Table S8) showed there was no collinearity between variables as shown by Heatmap (Fig. 3B).

Women aged 30–39 years and 40–49 years were significantly more likely to be screened compared to those aged 25–29 years, with aPRs of 1.57 (95% CI: 1.32–1.86) and 1.86 (1.58–2.20), respectively. Higher education was associated with increased screening uptake: women with tertiary education had an aPR of 1.36 (95% CI 1.09–1.68) compared to those with primary education or less (< = 7 years). Compared to Black African women, those identifying as White and of Mixed ancestry had higher screening uptake: aPRs of 1.25 (95% CI 1.07–1.45) and 1.44 (95% CI 1.26–1.64), respectively. Wealth was positively associated with screening where women in the highest wealth quintile had an aPR of 1.56 (95% CI 1.21–1.99) compared to those in the lowest. Being married was marginally associated with higher screening uptake (aPR: 1.12, 95% CI 1.00–1.24) (Table 2).

**Table 2:** Multivariable analysis of factors affecting cervical cancer screening among women aged 25–49 years in South Africa

Predictors	Model^a^ Sociodemographic	Model^b^ Female autonomy	Model^c^ Healthcare access	Model^d^ Risky lifestyle behaviors
Age group (years)	aPR (95% CI)	aPR (95% CI)	aPR (95% CI)	aPR (95% CI)
25–29	1.00 (ref)	1.00 (ref)	1.00 (ref)	1.00 (ref)
30–394	**1.57 (1.32–1.86)**	**1.56 (1.31–1.84)**	**1.55 (1.32–1.84)**	**1.56 (1.32–1.84)**
40–49	**1.86 (1.58–2.20)**	**1.86 (1.58–2.18)**	**1.85 (1.55–2.20)**	**1.82 (1.54–2.09)**
Education (years)				
Primary or less (< = 7 years)	1.00 (ref)	1.00 (ref)	-	-
Secondary (8–12)	1.19 (0.99–1.43)	1.18 (0.98–1.42)	-	-
Higher	**1.36 (1.09–1.68)**	**1.32 (1.06–1.63)**	-	-
Employment status				
No	-	1.00 (ref)	-	-
Yes	-	1.08 (0.97–1.20)	-	-
Population group				
Black	1.00 (ref)	1.00 (ref)	1.00 (ref)	1.00 (ref)
White	**1.25 (1.07–1.45)**	**1.26 (1.08–1.47)**	**1.28 (1.11–1.49)**	**1.22 (1.04–1.43)**
Mixed ancestry	**1.44 (1.26–1.64)**	**1.44 (1.27–1.63)**	**1.40 (1.23–1.58)**	**1.38 (1.21–1.56)**
Asian	1.05 (0.87–1.27)	1.06 (0.88–1.29)	1.06 (0.88–1.30)	1.03 (0.77–1.24)
Wealth index (quintiles)				
1 (lowest)	1.00 (ref)	1.00 (ref)	1.00 (ref)	1.00 (ref)
2	1.09 (0.90–1.31)	1.07 (0.89–1.29)	1.11 (0.92–1.33)	1.12 (0.86–1.34)
3	1.23 (1.00–1.51)	1.23 (1.00–1.50)	**1.24 (1.02–1.51)**	**1.30 (1.07–1.57)**
4	**1.37 (1.09–1.73)**	**1.36 (1.09–1.69)**	**1.34 (1.08–1.67)**	**1.46 (1.20–1.78)**
5 (highest)	**1.56 (1.21–1.99)**	**1.52 (1.22–1.92)**	**1.51 (1.20–1.91)**	**1.76 (1.46–2.11)**
Marital status				
Not married	1.00 (ref)	-	-	-
Married	1.12 (1.00–1.24)	-	-	-
Other	1.05 (0.89–1.24)	-	-	-
#Women empowerment status				
Not empowered	-	1.00 (ref)	-	-
Less empowered	0.94 (0.79–1.11)	0.94 (0.79–1.11)	-	-
Highly	-	**1.16 (1.04–1.30)**	-	-
Place of residence				
Urban	-	-	1.00 (ref)	-
Rural	-	-	0.96 (0.82–1.12)	-
Health insurance coverage				
No	-	-	1.00 (ref)	-
Yes	-	-	**1.27 (1.12–1.45)**	-
HIV tested ever				
No	-	-	1.00 (ref)	-
Yes	-	-	**1.79 (1.40–2.30)**	-
Regular use of contraceptive				
No	-	-	1.00 (ref)	-
Oral/injectable	-	-	1.13 (1.00–1.28)	-
Other	-	-	1.12 (0.99–1.27)	
Parity				
0–1	-	-	1.00 (ref)	-
2	-	-	1.03 (0.90–1.17)	-
3 +	-	-	0.97 (0.82–1.13)	-
Having children aged < 5 years				
No	-	-	1.00 (ref)	-
Yes	-	-	0.90 (0.81–1.01)	-
Smoking frequency				-
Never or occasional	-	-	-	1.00 (ref)
> = 1 cigarette per day	-	-	-	0.95 (0.81–1.12)
Alcohol misuse			-	
No (CAGE = 0–1)	-	-	-	1.00 (ref)
Yes (CAGE > = 2)	-	-	-	1.23 (1.10–1.38)
Number of sexual partners			-	
0	-	-	-	1.00 (ref)
1	-	-	-	0.94 (0.84–1.04)
2 +	-	-	-	0.96 (0.71–1.30)

^a^Basic model: variables included were age(years), place of residence, population group, wealth index (quantile)

^b^Variables included in the model were marital status, women empowerment status, employment, education (years) and adjusted for the basic model

^c^Independent variables included in the model were HIV tested ever, distance as a barrier to health clinic, health insurance coverage, regular use of contraceptive, parity and having children aged < 5 years while adjusting for the basic model

^d^Independent variables included in the model were smoking frequency, alcohol use and number of sexual partners while adjusting for the basic model

The results statistically signifcant at *p*<0.05

**Fig. 4:**
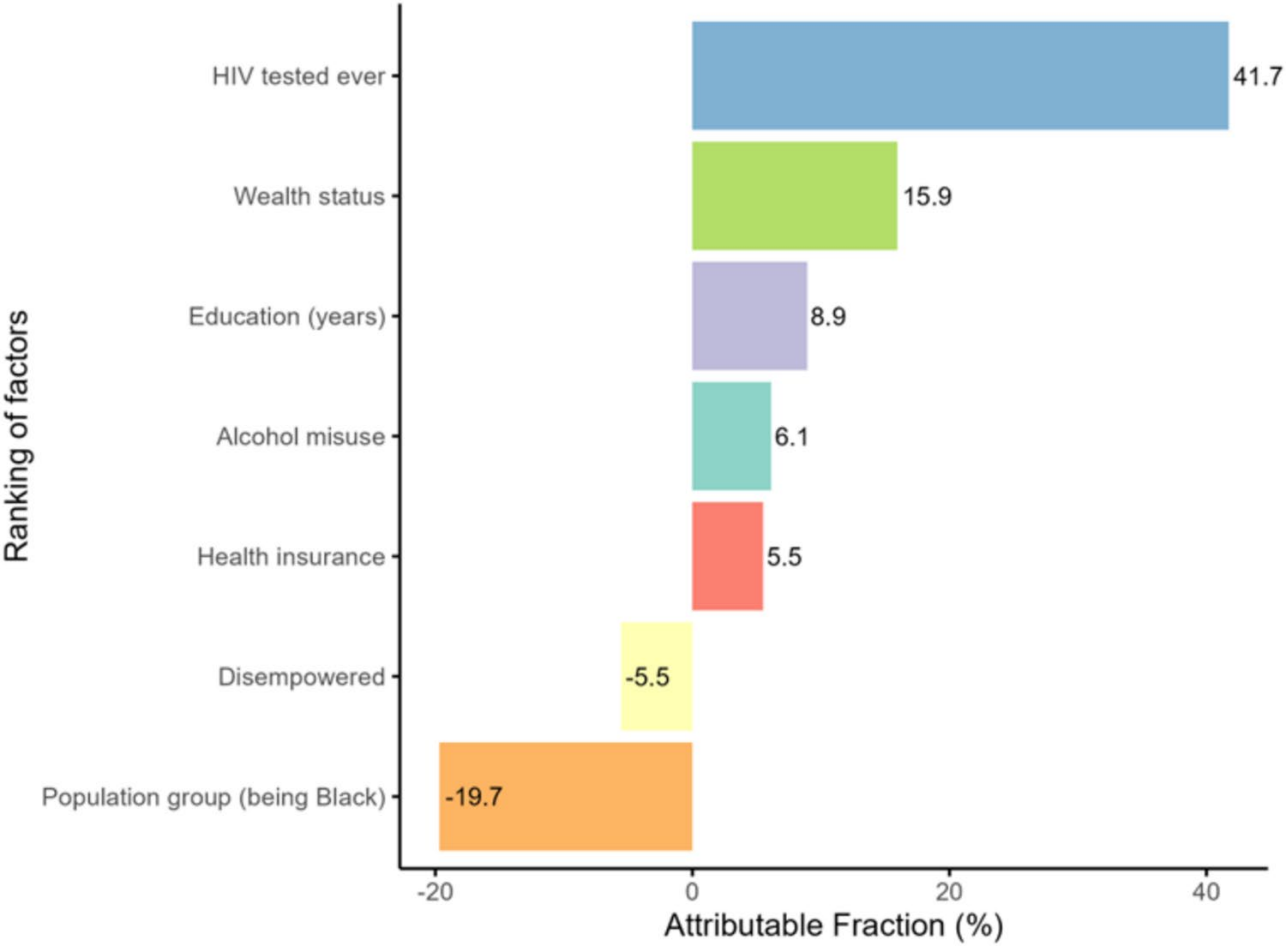
Ranking of factors in order of importance for cervical cancer screening in South Africa

Highly empowered women had significantly higher screening uptake compared to those not empowered (aPR: 1.16, 95% CI 1.04–1.30). The effect of higher education adjusted for autonomy was slightly attenuated but remained significant (aPR: 1.32, 95% CI 1.06–1.63). The associations for age, population group, and wealth status remained stable (Table 2). Those who had ever tested for HIV were nearly twice as likely to have been screened for cervical cancer (aPR: 1.79, 95% CI 1.40–2.30). Compared to no contraceptive use, regular use of oral/injectable contraceptives was marginally associated with increased screening (aPR: 1.13, 95% CI 1.00–1.28). Women with health insurance had significantly higher screening uptake (aPR: 1.27, 95% CI 1.12–1.45) (Table S2) The strength of associations for adjusted age, population group, and wealth status remained across all the models (Table 2). Women who reported alcohol misuse had significantly higher screening uptake (aPR: 1.23, 95% CI 1.10–1.38) (Fig. 4). HIV testing was ranked first ( AF=41.7%), followed by population group (being Black) (AF=19.7%), wealth index (AF=15.9%), and educational attainment (AF=8.9%). In contrast, alcohol misuse (AF=6.1%), having health insurance (AF=5.5%) and disempowerment (AF=5.5%) whad lowest attributable fractions.

### Sensitivity analysis

In the sensitivity analysis, which included all covariates simultaneously, the associations observed in the primary models remained robust. Women aged 30–39 years and 40–49 years continued to demonstrate significantly higher screening uptake compared with those aged 25–29 years (aPR: 1.57, 95% CI: 1.34–1.85 and aPR: 1.91, 95% CI: 1.64–2.23, respectively). The strength of association for population group and wealth quintile was slightly attenuated but remained significant, for Mixed ancestry women (aPR: 1.36, 95% CI 1.19–1.54) and White women (aPR: 1.19, 95% CI 1.03–1.39) more likely to be screened than Black African women, and those in the highest wealth quintile more likely to be screened than those in the lowest (aPR: 1.43, 95% CI 1.15–1.80). Notably, the effect of wealth was reduced compared to earlier models, suggesting partial mediation by healthcare access and autonomy. Highly empowered women (aPR: 1.14, 95% CI 1.02–1.27), those with health insurance (aPR: 1.21, 95% CI 1.07–1.37), and those who had ever tested for HIV (aPR: 1.76, 95% CI 1.38–2.26) were significantly more likely to be screened for cervical cancer. Alcohol misuse also remained independently associated with increased screening uptake (aPR: 1.19, 95% CI 1.07–1.32). These findings confirm the robustness of key predictors while highlighting the complex interplay between sociodemographic, behavioral, and healthcare access factors (Table S3).

## Discussion

This study provides a comprehensive, multifactorial assessment of factors associated with cervical cancer screening in South Africa. This contrasts with previous studies which mainly analyzed the risk factors in silos rather than providing a more integrated assessment of multiple determinants. Overall, cervical cancer screening uptake was suboptimal (< 50%), remaining below the South African Department of Health and WHO target of 70% coverage. In contrast, Phaswana-Mafuya and Peltzer [15] reported a higher Pap smear prevalence of 52.0%, which may reflect differences in sample size and age range across studies. Cervical cancer screening differed significantly by population group, socioeconomic status, and education level. Screening was higher among White and Mixed ancestry compared to Black African women, and among wealthier and more educated individuals, i.e., those with secondary and tertiary education.

HIV testing history was strongly associated with increased screening uptake and ranked the main key factor for cervical cancer screening in South Africa. The strong association of HIV testing with cervical cancer screening suggests that screening may often occur opportunistically. Other studies from sub-Saharan Africa [25–27] also report an association between HIV testing history and cervical cancer screening. Women who engage more frequently with healthcare services may be more likely to undergo screening for cervical cancer.

Black African women had lower cervical cancer screening uptake in South Africa, and this was the second most important factor associated with screening. This finding is consistent with studies from South Africa, Great Britain, and the United States that reported disparities in cervical cancer screening between Black and White populations [15, 26, 27]. Our findings suggest that expanding the health workforce, implementing task shifting, and training community health workers to organize and mobilize communities for outreach services could help improve screening uptake for cervical cancer.

Socioeconomic disparities remain a central barrier to equitable screening access [10, 32]. In our study wealth status was strongly associated with cervical cancer screening uptake and ranked as the third most important factor. Women in the wealthier quintile were more likely to be screened than those in the lowest quintile because of easier access to healthcare and were more likely to afford payments of healthcare costs associated with healthcare visits i.e., transport costs and hospital bills, similar to findings reported globally [23, 33–37]. Implementation of screening in the workplace and flexible screening times with mobile clinics in the communities over the weekends, etc. would improve cervical cancer screen uptake among women with low socioeconomic status. Furthermore, implementing educational initiatives to enhance public awareness of the importance of cervical cancer prevention and screening represents a crucial strategy for tackling this public health challenge.

Higher levels of education are generally associated with improved knowledge, awareness and better financial incomes which in turn facilitate active participation in cervical cancer prevention efforts [28–30]. In our study, higher education attainment was associated with cervical cancer screening uptake in South Africa, and it was ranked as the fourth most important determinant of cervical cancer screening uptake. Other studies, in agreement with our findings, have reported the association of secondary or higher education levels with an increase in uptake of screening for cervical cancer [15, 25, 30–32]. Of concern, there were observed disparities in the levels of education attainment by population groups with White women having higher levels of education compared to Black women (Table S2). Developing media campaigns (billboards, radio and TV) in the local language tailored cervical cancer screening could enhance knowledge and awareness of cervical cancer among Black communities.

The association between alcohol misuse and increased cervical cancer screening uptake as reported in this study is equivocal in the literature and contributed least to cervical cancer screening. While some studies from The Netherlands and Norway have shown an association between high alcohol consumption and cervical cancer screening [33, 34], other studies have not [35]. Although counterintuitive, this may reflect increased contact with health services for screening conditions associated with alcohol misuse leading to poor health and more frequent clinic attendances, thereby creating opportunities for opportunistic screening [36]. It is possible that women in our study who misused alcohol may have been involved in other risky sexual behaviors such as having multiple sexual partners which increases the risk of contracting human papilloma virus (HPV) and is linked to cervical cancer. Alternatively, residual confounding. Further research is needed to understand this association.

Our findings showed that having health insurance was strongly associated with cervical cancer screening uptake in South Africa but was ranked the least important determinant. Similar findings were observed in other South African studies, and in Latin America and USA [15, 37, 38]. However, Adonis et al.[39] in South Africa found that being on health insurance alone was insufficient to increase screening uptake. Screening behavior was strongly influenced by plan type and participation in incentive programs, highlighting structural and behavioral factors not just insurance drive utilization. It is important to note that in our study White compared to Black women were more likely to be covered by health insurance, suggesting that socioeconomic status may significantly affect an individual’s health by limiting access to health insurance and healthcare services [38, 40]. These findings highlight the need for better healthcare access in the targeted communities, particularly those delivered through community outreach as a means of improving screening uptake.

While an association between women empowerment and the uptake in cervical cancer screening was demonstrated, being disempowered was the least important factor for cervical cancer screening in this study. Our findings are in agreement with those from other low- and middle-income countries including Lesotho, Tanzania and Nepal [14, 20, 41] suggesting that empowering women would provide the opportunity to make informed health decisions. In the context of cervical cancer screening, this may translate to greater screening uptake and control over other health-related activities which may yield substantial health dividends [12, 47].

Of note, in other studies, engagement of male partners has been shown to significantly influence women’s decisions to undergo cervical cancer screening [42]. However, in our study being married was marginally associated with higher screening uptake and currently married women showed higher screening rates in older age groups (especially 40–49). Being married may also translate into access to more funds for travel to clinics, and if married women were not working, they would not be so time constrained/concerned about loss of income from missing work. These findings suggest that partners’ support in health-seeking behavior or male involvement in cervical cancer screening may encourage women to attend cervical cancer screening [34, 42, 43].

Although in our study regular use of oral/ injectable contraceptive was marginally associated with uptake for cervical screening. However, a study in Norway and Kenya found a significant association between regular use of contraceptives and cervical cancer screening [34, 44]. Women who engage in regular use of contraceptive services may exhibit greater health consciousness than those who do not, potentially leading to a higher propensity for seeking preventive healthcare services and screening [34]. Thus, postnatal and immunization clinics at primary health centers may serve as strategic entry points to enhance awareness and uptake of cervical cancer screening.

The greater likelihood for cervical cancer screening with older age in this study is in keeping with reports from previous studies [15, 34, 45, 46]. This may be attributable to greater engagement with healthcare services among older women because of poorer health compared with younger women or because of more opportunities for screening due to their longevity [36, 47].

### Strengths and limitations

This study’s strengths include the large sample size, and that the data are nationally representative, which enable the generalizability of the findings to all women in South Africa. Another strength is that all statistical analyses included survey weights to account for the complex sampling design and to ensure accurate estimation of population-level means. The population-based design and the use of GLMs with a Poisson distribution and log-link function to estimate aPR provided close estimates of the real-world cervical cancer screening uptake in South Africa. By analyzing these variables in a unified manner and prioritizing them by means of risk and numbers of screened, our findings reveal a more nuanced assessment of sociodemographic, healthcare access women, empowerment and lifestyle variables that shape cervical cancer screening behavior in South Africa. In addition, because of our approach, our findings add to the growing body of evidence on cervical cancer prevention with a specific focus on South Africa. However, the cross-sectional nature of the data limits causal inferences, and some associations may be subject to reverse causality or residual confounding. We used the 2016 SADHS data which might not reflect the current screening behaviors of women in South Africa. The SADHS asked women only about Pap smear uptake without asking other modalities of screen, i.e., (VIA, HPV DNA). Additionally, screening history was self-reported, which may introduce recall or social desirability bias. However, the consistency of findings across multiple indicators and the alignment with existing literature suggest that any such bias is unlikely to have significantly distorted the observed patterns. Notably, despite its conceptual importance, the empowerment tool applied in our study was developed and tested in the Black population of Lesotho [45]; therefore, it might have limitations in other settings. This may likely reflect both measurement limitations where empowerment was operationalized using proxy indicators and the possibility that its effects were mediated through more proximal socioeconomic factors such as transport costs, distance to clinics, and long waiting times at the clinics. Although trends were not statistically significant, they may indicate underlying disparities in screening behavior that merit further investigation into larger or more adequately powered studies. In culturally sensitive settings, individual-level empowerment may be insufficient to overcome health-systems barriers to screening, resulting in a diminished direct impact in statistical models. Finally, while the study captures a wide range of determinants, unmeasured contextual or cultural factors may also influence screening behavior and warrant further qualitative exploration.

### Recommendations

Increasing healthcare workforce by hiring and educating a lot of community health surveillance assistants. Expanding community-based outreach for across population groups affected are essential steps toward achieving equitable coverage. Intensifying community education for cervical cancer screening and awareness program across population groups to increase health literacy would also assist in reducing racial disparities gaps current present in the screening program. Leveraging existing infrastructure such as office screening, mobile clinics to deliver comprehensive women’s health services represents a pragmatic and scalable approach to improving coverage.

## Conclusions

In South Africa, Pap smear uptake remains suboptimal, particularly among Black women. There is a need for enhanced awareness and education initiatives as well as novel strategies involving community-based screening programs to encourage the uptake of cervical screening.

## Supplementary Information

Below is the link to the electronic supplementary material.

Supplementary file1 (DOCX 96 KB)

## Data Availability

The study used secondary data from dhsprogram.org, which is publicly available at https://dhsprogram.com/data/available-datasets.cfm.
