# Supplementary material for: Factors associated with cervical cancer screening in the South African demographic and health survey
Source: Cancer Causes Control. 2026 Jun 30;37(7):113. doi: 10.1007/s10552-026-02205-5 (PMC13319914; doi:10.1007/s10552-026-02205-5)
Supplement: Supplementary file 1 — Supplementary file1 (DOCX 96 KB) [file 10552_2026_2205_MOESM1_ESM.docx]

**Supplementary Information**

Table S 1: Modified Poisson regression with survey weights evaluating the joint effect of wealth index and race, adjusted for age, on Pap smear screening uptake.

| **Predictor** | **aPR (95%)** | **p-value** |
| --- | --- | --- |
| **Age group (years)** |  |  |
| 25-29 | **1.00(reference)** | **-** |
| 30-394 | **1.57 (1.33-** | **<0.001** |
| 40-49 | **1.80 (1.52-** | **<0.001** |
| **Population group** |  |  |
| Black | **1.00(reference)** | **-** |
| White | **0.00 (0.00-0.001)** | **<0.001** |
| Mixed ancestry | **1.76 (1.16-2.69)** | **<0.009** |
| Indian/Asian | 1.04 (0.83-1.30) | **0.7540** |
| **Wealth index (quintiles)** |  |  |
| 1 (lowest) | **1.00(reference)** |  |
| 2 | **1.13 (0.93-1.37)** | **0.220** |
| 3 | **1.34 (1.09-1.64)** | **0.005** |
| 4 | **1.49 (1.20-1.85)** | **<0.001** |
| 5 (highest) | **1.83 (1.50-2.24)** | **<0.001** |
| Race (White) Wealth index |  |  |
| Quantile3x White | **69730.85 (5066.97-959625.89)** | **<0.001** |
| Quantile4x White | **188159.00 (25625.72-1381573.13)** | **<0.001** |
| Quantile5x White | **164233.34 (22720.05-1187171.49)-** | **<0.001** |

Table S 2: Attributable Fraction of the risk factors for cervical cancer screening

| Predictor | Screened | AFE | Attributable number | AF (%) |
| --- | --- | --- | --- | --- |
| **Sociodemographic** |  |  |  |  |
| **Age group (years)** |  |  |  |  |
| 25-29 | 216 |  |  |  |
| 30-394 | 535 | 0.363 | 194.2 |  |
| 40-49 | 511 | 0.462 | 236.3 |  |
| **Education (years)** |  |  |  | 8.9 |
| Primary or less (<=7 years) | 133 |  |  |  |
| Secondary (8-12) | 505 | 0.160 | 80.6 |  |
| Higher | 623 | 0.265 | 164.9 |  |
| **Population group** |  |  |  |  |
| Black | 982 | -0.253 | -248.6 | -19.7 |
| White | 81 |  |  |  |
| Mixed ancestry | 167 | 0.200 | 16.2 |  |
| Indian/Asian | 32 | 0.306 | 51.2 |  |
| **Wealth index (quintiles)** |  |  |  | 15.9 |
| 1 (lowest) | 158 |  |  |  |
| 2 | 187 | 0.083 | 15.4 |  |
| 3 | 257 | 0.187 | 48.1 |  |
| 4 | 287 | 0.270 | 77.5 |  |
| 5 (highest) | 370 | 0.359 | 132.8 |  |
| **Women autonomy** |  |  |  |  |
| **Women empowerment status** |  |  |  | -5.5 |
| Not empowered | 622 |  |  |  |
| Less empowered | 140 | -0.064 | -8.9 |  |
| Highly | 493 | 0.138 | 68.8 |  |
| **Healthcare access** |  |  |  |  |
| **Health insurance coverage** |  |  |  | 5.5 |
| No | 935 |  |  |  |
| Yes | 326 | 0.213 | 69.3 |  |
| **HIV tested ever** |  |  |  | 41.7 |
| No | 69 |  |  |  |
| Yes | 1192 | 0.441 | 526.1 |  |
| **Lifestyle behaviours** |  |  |  |  |
| **alcohol misuse** |  |  |  | 6.1 |
| No (CAGE =0-1) | 847 |  |  |  |
| Yes (CAGE>=2) | 414 | 0.187 | 77.4 |  |

Table S 3: Sensitivity analysis an All-inclusive model

| Predictor | aPR (95% CI) |
| --- | --- |
| **Sociodemographic** |  |
| **Age group (years)** |  |
| 25-29 | 1.00 (ref) |
| 30-394 | **1.57 (1.34-1.85)** |
| 40-49 | **1.91 (1.64-2.23)** |
| **Education (years)** |  |
| Primary or less (<=7 years) | 1.00 (ref) |
| Secondary (8-12) | 1.13 (0.93-1.35) |
| Higher | 1.21 (0.93-1.49) |
| **Population group** |  |
| Black | 1.00 (ref) |
| White | **1.19 (1.03-1.39)** |
| Mixed ancestry | **1.36 (1.19-1.54)** |
| Indian/Asian | 1.07 (0.88-1.31) |
| **Wealth index (quintiles)** |  |
| 1 (lowest) | 1.00 (ref) |
| 2 | 1.07 (0.89-1.29) |
| 3 | 1.20 (0.99-1.46) |
| 4 | **1.29 (1.05-1.60)** |
| 5 (highest) | **1.43 (1.15-1.80)** |
| **Women autonomy** |  |
| **Women empowerment status** |  |
| Not empowered | 1.00 (ref) |
| Less empowered | 0.92 (0.77-1.09) |
| Highly | **1.14 (1.02-1.27)** |
| **Healthcare access** |  |
| **Health insurance coverage** |  |
| No | 1.00 (ref) |
| Yes | **1.21 (1.07-1.37)** |
| **HIV tested ever** |  |
| No | 1.00 (ref) |
| Yes | **1.76 (1.38-2.26)** |
| **Lifestyle behaviours** |  |
| **alcohol misuse** |  |
| No (CAGE =0-1) | 1.00 (ref) |
| Yes (CAGE>=2) | **1.19 (1.07-1.32)** |

Table S 4: Prevalence of education and insurance coverage by population group among cervical cancer screened women.

| **Variable** | **Population group** | | | | | |
| --- | --- | --- | --- | --- | --- | --- |
|  | Total (N=1,261) | Black  n (%) | White  n (%) | Mixed ancestry  n (%) | Indian/Asian  n (%) | **p-value** |
| **Education** |  |  |  |  |  | **<0.001** |
| Primary or less (<=7 years) | 132 | 109 (11.1) | 1 (0.6) | 20 (12.1) | 3 (9.2) |  |
| Secondary (8-12) | 505 | 409 (42.7) | 5 (5.6) | 82 (49.5) | 10 (32.0) |  |
| Higher | 623 | 464 (47.3) | 76 (93.7) | 65 (38.9) | 18 (58.7) |  |
| **Insurance** |  |  |  |  |  | **<0.001** |
| No | 935 | 785 (79.9) | 17 (21.6) | 117 (70.5) | 17 (50.4) |  |
| Yes | 326 | 198 (20.1) | 63 (78.4.0) | 50 (30.0) | 15 (49.6) |  |

Table S 5: Proportion of empowerment by population group

|  | Empowerment | | p-value |
| --- | --- | --- | --- |
| **Population group** | Not empowered  n (%) | Empowered  n (%) | **<0.001** |
| Black | 1,437 (60.7) | 930 (39.3) |  |
| White | 24 (28.6) | 60 (71.4) |  |
| **Wealth status** |  |  | **<0.001** |
| Poor | 68 (61.1) | 434 (38.9) |  |
| Rich | 511 (51.6) | 479 (48.4) |  |

Table S 6: Screening uptake by marital status across different age groups

|  | Age | | | | |
| --- | --- | --- | --- | --- | --- |
| **Marital status** | Total (N=1,261) | 25-29  n (%) | 30-39  n (%) | 40-49  n (%) | **p-value** |
| Never married | 484 | 106 (55.8) | 221 (42.1) | 157 (30.8) | <**0.001** |
| Currently | 621 | 77 (40.5) | 268 (51.1) | 276 (54.2) |  |
| Other | 119 | 7 (3.7) | 36 (6.9) | 76 (14.9) |  |

Table S 7: The Proportional of disempowered by population group

| Population Group | Total  N | Disempowered  n (%) | Empowered  n (%) | p-value |
| --- | --- | --- | --- | --- |
| Black | 2396 | 1420 (59.3) | 976 (40.7) | <0.001 |
| White | 105 | 27 (25.3) | 79 (76.7) |  |
| Mixed ancestry | 239 | 92 (38.4) | 147 (61.6) |  |
| Indian/Asian | 50 | 14 (27.5) | 36 (72.6) |  |

Table S 8: VIF results for checking of collinearity between predictors

| Predictor | VIF |
| --- | --- |
| Empowerment | 1.02 |
| Population group | 1.07 |
| Wealth status | 1.05 |


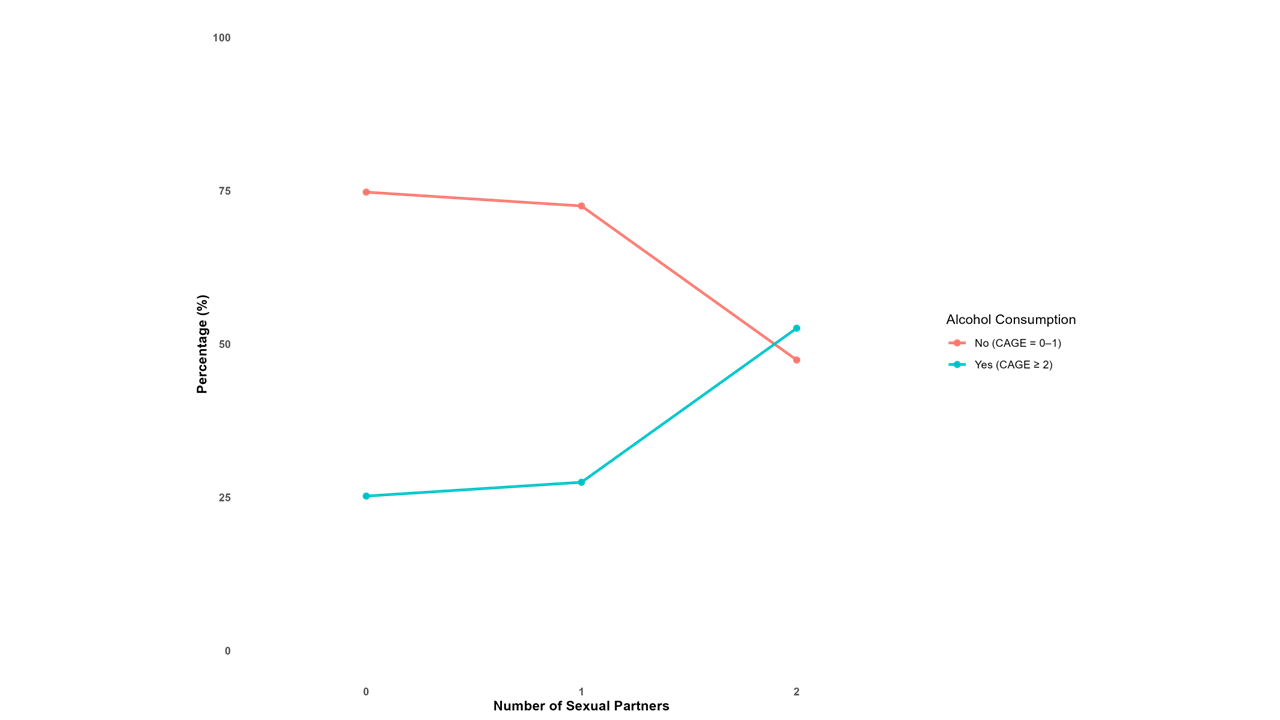


Fig. S1: Prevalence of Alcohol misuse by number of sexual partners in women reporting cervical screening.
